# Theoretical investigation of the electronic structure and magnetic properties of Ru doped LiFeAs

**DOI:** 10.1038/s41598-025-20774-4

**Published:** 2025-10-22

**Authors:** Manza Zityab Kasiab, Kumneger Tadele, Mesfin Asfaw Afrassa, Omololu Akin-Ojo, Tesfaye Feyisa Hurrisa

**Affiliations:** 1 Department of Applied Physics, College of Applied Natural Science, Adama Science and Technology University, P.O.Box 1888, Adama, Ethiopia; 2 Department of Physics, University of Ibadan, Oyo, Nigeria

**Keywords:** Density functional theory, Electronic structure, Density of states, Phonon dispersion frequency, Fermi surface, Materials science, Physics

## Abstract

This study performed first-principles calculations using Density Functional Theory (DFT) and DFT+*U* within the Quantum-Espresso package. . The electronic structure and magnetic properties of Ru-doped LiFeAs were systematically analyzed at doping concentrations of 25%, 50%, and 100%, revealing significant modifications induced by Ru substitution. The optimized lattice parameter of pristine LiFeAs is 3.767 Å, in excellent agreement with the experimental value of 3.77 Å. Upon 25% Ru substitution, the lattice parameter expands slightly to 3.786 Å, reflecting the structural response to partial Ru incorporation. The computed electronic structure and magnetic properties of LiFe_1−x_Ru_x_As confirm its metallic nature, with no detectable band gaps. Density of States (DOS) calculations reveal that the conduction band near the Fermi level is primarily dominated by Fe-3*d* and Ru-4*d* orbitals, while the valence band is largely influenced by As-*p* states. With 25% Ru substituted, the electronic band structure shows a strong buildup of states close to the Fermi level, suggesting that the material is becoming more metallic. This elevated electronic density at the Fermi surface is likely to have a substantial impact on the material’s superconducting behavior and charge transport properties, potentially enhancing its conductivity and modifying the electron pairing interactions. In the ferromagnetic (FM) configuration, Ru doping enhances both spin polarization and metallicity, whereas the antiferromagnetic (AFM) state exhibits a suppressed DOS near the Fermi level. The inclusion of the Hubbard *U* correction provides improved insight into localized electron interactions, particularly in the Fe 3*d* orbitals. This study contributes to a deeper understanding of the interplay between doping, electronic correlation, and magnetism in iron-based superconductors. The pristine, 25, and 50% Ru-doped LiFeAs systems retain AFM coupling, while full (100%) Ru substitution induces a transition to a nonmagnetic state. The magnetic moments of Fe atoms decrease progressively with increasing Ru concentration, indicating a suppression of magnetism.

## Introduction

Iron-based superconductors have become a central focus in condensed matter physics since the groundbreaking discovery of superconductivity in LaFeAsO$$\phantom{0}_{1-x}$$F$$\phantom{0}_{x}$$, which exhibited a transition temperature (T$$\phantom{0}_{c}$$) of 26K in 2008^1^. These materials possess layered crystal structures composed of FeAs or FeSe planes, where superconductivity emerges from intricate interactions involving spin fluctuations and multi-orbital correlations. Iron-based superconductors are generally classified into distinct families, including the ‘1111’ (RFeAsO, R = rare earth), ‘122’ (BaFe$$\phantom{0}_{2}$$As$$\phantom{0}_{2}$$), ‘111’ (LiFeAs), and ‘11’ (FeSe) systems^2^. Notably, compounds such as SmFeAsO$$\phantom{0}_{1-x}$$F$$\phantom{0}_{x}$$ have achieved superconducting transition temperatures (T$$\phantom{0}_{c}$$) above 55K under optimal doping and synthesis conditions^3,4^. Iron-based superconductors have attracted considerable interest for their unconventional superconductivity and rich magnetic behavior. Among them, LiFeAs, a representative of the 111-type iron pnictides, is particularly notable as it exhibits superconductivity in its stoichiometric form, without requiring external doping or pressure, with a transition temperature of approximately 18K^5^. Its simple crystal structure and lack of magnetic ordering under ambient conditions provide an excellent model system for both theoretical and experimental exploration of the interplay between superconductivity and magnetism in Fe-based materials^6^. LiFeAs also serves as a fundamental reference system for studying the effects of transition metal doping. In particular, substitution of Fe with Ru has attracted attention due to the isoelectronic nature of Ru and its ability to influence the electronic structure, magnetic exchange interactions, and structural properties without altering the charge carrier concentration^7,8^. Experimental techniques such as angle-resolved photoemission spectroscopy (ARPES), nuclear magnetic resonance (NMR), and neutron scattering have been widely applied to probe the multiband nature and superconducting gap symmetry of LiFeAs^9,10^. These works reveal that the compound exhibits multiple hole and electron pockets at the Fermi surface, and its superconductivity likely arises from spin-fluctuation-mediated pairing rather than conventional electron-phonon interaction^11^. From a theoretical perspective, DFT studies have confirmed the absence of magnetic ordering in the ground state of LiFeAs, in contrast to other iron-based superconductors like BaFe$$\phantom{0}_{2}$$As$$\phantom{0}_{2}$$^12,13^. Doping of LiFeAs with 4*d* elements such as Ru introduces minimal charge doping but leads to significant changes in the structural and magnetic properties^14^. The substitution of Ru at the Fe site increases lattice parameters and induces subtle modifications in the Fe–As bond angle, which are crucial to the emergence and tuning of magnetic interactions and superconductivity^15^. These findings establish a strong foundation for investigating the effect of Ru substitution using first-principles calculations. Our work complements these studies by analyzing spin-resolved PDOS and band structures across different magnetic configurations and Ru concentrations. The Leibniz Institute for Solid State and Materials Research Dresden (IFW Dresden) conducted research in 2010 that revealed additional intriguing characteristics of 111-type iron-based superconductor^16^. The 111-type iron-based superconductor AFeM (A = Li, Na; M = As, P)^17^ has attracted significant attention because of its unique structural and physical properties. Unlike other iron-based superconductors, the 111-type materials feature a more straightforward crystal structure without spacer layers, making them ideal for theoretical modeling and experimental studies^18^. LiFeAs is considered an ideal platform for testing theories of unconventional superconductivity due to its intrinsic superconducting state without the need for chemical doping, along with its moderate electron correlations and pronounced multiband electronic structure^19,20^. The interplay between superconductivity and magnetism in LiFeAs has been extensively studied, with results suggesting that doping and substitution play crucial roles in tuning the electronic and magnetic properties of LiFeAs^21^. Beyond superconductivity, Ru substitution in Fe$$\phantom{0}_{2}$$O$$\phantom{0}_{3}$$ and Bi$$\phantom{0}_{12}$$TiO$$\phantom{0}_{20}$$ enhance optical absorption, with applications in biosensors and photonics^22^. Structural modifications, particularly changes in lattice parameters, Fe–As bond lengths, and bond angles induced by Ru substitution, play a crucial role in tuning the delicate interplay between superconductivity and magnetism in iron-based superconductors^23^. These subtle distortions can significantly influence electronic bandwidth, orbital hybridization, and magnetic exchange interactions, thereby impacting the superconducting ground state. The precise role of magnetic fluctuations and the pairing mechanism in LiFeAs is still debated^24^, while pristine LiFeAs lacks magnetic ordering, doping with the transition metal Ru imparts magnetic properties, thereby offering insights into the magnetic interactions that coexist or compete against superconductivity^25^. Doping transition metals into the Fe site is a widely used strategy to probe the evolution of electronic structure, magnetism, and superconducting behavior in iron-based superconductors (IBSCs). Among various dopants, Ru is of particular interest due to its isovalent substitution character and larger ionic radius compared to Fe, which allows it to modulate the electronic bandwidth, lattice parameters, and magnetic fluctuations without significantly altering the carrier concentration^26,27^. Ru substitution has been reported to suppress magnetic ordering while preserving or slightly modifying the superconducting state in several iron pnictides^28–30^. More recently, computational approaches have been increasingly employed to predict novel superconductors with remarkably high transition temperatures, as demonstrated by the discovery of Mg–Zr–H hydrides with T$$\phantom{0}_{c}$$ up to 214.3 K^31^. Although Ru substitution has been widely investigated in other iron pnictides for instance, in BaFe$$\phantom{0}_{2-x}$$Ru$$\phantom{0}_{x}$$As$$\phantom{0}_{2}$$, where bulk superconductivity emerges near T$$\phantom{0}_{c}$$ = 22 k with concurrent suppression of the magnetic transition^32^, and in SrFe$$\phantom{0}_{2-x}$$Ru$$\phantom{0}_{x}$$As$$\phantom{0}_{2}$$ , which exhibits superconductivity up to T$$\phantom{0}_{c}$$ = 13.5 K at x = 0.7^33^. Unlike Ba- and Sr-based 122 compounds, LiFeAs exhibits superconductivity in its stoichiometric form without the need for external doping or applied pressure, making it an ideal platform to disentangle the intrinsic effects of Ru substitution from those arising due to extrinsic tuning parameters. First-principles studies suggest that Ru incorporation in LiFeAs primarily alters the electronic environment by broadening bandwidths, reshaping the Fermi surface, and suppressing magnetic ordering, rather than through simple carrier doping^34^. Furthermore, experimental and theoretical works have shown that Ru substitution can drive subtle yet significant structural changes, including lattice expansion and modifications of the Fe–As bond geometry, both of which critically influence the electronic structure and superconducting mechanism in iron pnictides^35^  In particular, Reticcioli et al.^36^, using DFT+U calculations on BaFe$$\phantom{0}_{2}$$As$$\phantom{0}_{2}$$ and LaFeAsO, demonstrated that structural distortions induced by Ru substitution dominate the resulting changes in electronic structure and magnetic properties, outweighing simple charge-transfer effects. Taken together, the﻿ insights underscore the pivotal role of lattice and structural effects in shaping the physics of Ru-doped iron-based superconductors, while highlighting the absence of a comprehensive theoretical understanding for the case of LiFeAs. Similarly, Lohani et al.^37^ applied the LDA+*U* method to investigate correlation effects in FeSe and FeTe, finding that Coulomb interactions and Hund’s coupling strongly influence the electronic structure. Their results revealed orbital-selective correlations arising from interorbital hybridization among Fe-3*d* orbitals mediated by chalcogen p orbitals, underscoring the complex interplay between electron correlations and orbital physics in these systems. Building on this, our study employs first-principles calculations within both DFT and DFT+*U* frameworks to systematically investigate pristine LiFeAs and its Ru-doped variants at 25%, 50%, and 100% substitution levels, considering FM, AFM, and NM configurations. The inclusion of on-site Coulomb interactions in the DFT+*U* scheme proves essential for reproducing the structural properties with high fidelity.$$\mathring{A}$$ $$\mathring{A}$$$$\Delta$$$$_{c}^{(MFA)}$$$$_{c}(k)^{(corr)}$$

## Computational detail

The electronic structure calculations were performed using DFT and its extension DFT+U to account for on-site electron correlation effects. The computations were carried out with the Quantum-Espresso code using the Perdew-bURKE-Ernzerhof (PBE) correlation function in DFT as well as projector-augmented wave (PAW) pseudopotential ^38^. Both magnetic and non-magnetic configurations were examined to investigate the influence of Ru substitution on the electronic and magnetic properties of LiFeAs. To gain detailed insights into the electronic structure, calculations of the DOS (Partial DOS), phonon dispersion, and Fermi surface topology were performed for pristine and Ru-doped LiFeAs. Doping levels of x = 0.25, 0.50, and 1.00 in the composition LiFe$\phantom{0}_{1-x}$Ru$\phantom{0}_{x}$As were considered to systematically study the evolution of the properties with increasing Ru content. Magnetic moment calculations were performed to assess the influence of Ru doping on magnetization. The structural model was based on the tetragonal LiFeAs superconductor, which crystallizes in the P4/nmm space group, with a lattice parameter of 3.76 Å. The plane-wave energy and charge density cutoffs were set to 816 eV and 6528eV, respectively. In this study, the on-site Coulomb interaction parameter for Fe was determined to be approximately U = 5.0 eV using the linear response approach, a widely accepted method for estimating effective interaction parameters in correlated systems^47^ This value is consistent with the previously reported range of *U* = 2.5–6.0 eV for Fe in iron-based superconductors^48^ and effectively captures the correlation effects associated with the Fe 3d orbitals. The use of this U value ensures consistency with prior theoretical models and enables a reliable description of the correlated Fe 3d electrons in both pristine and doped systems. When combined with the DFT+*U* correction, this framework provides an improved treatment of electron localization, magnetic behavior, and the electronic structure near the Fermi level, while maintaining computational efficiency and accuracy. Brillouin zone sampling was carried out via an 8 $\times$ 8 $\times$ 6 Monkhorst-Pack k-point grid. The experimental lattice parameters for tetragonal LiFeAs are a = 3.77 Å and c = 6.36 Å^42^. Electronic band structures were computed along the high-symmetry directions $\Gamma$-X-M-$\Gamma$-Z-A-R-Z within the conventional tetragonal Brillouin zone^43^..

## Results and discussion

## Crystal structure

A systematic convergence test for lattice parameters, k-point sampling, and plane-wave cutoff energy was performed to ensure the reliability of the structural optimization. The optimized crystal structure of LiFeAs shows bond lengths and bond angles in reasonable agreement with experimental reports, although minor deviations are observed. Specifically, the calculated bond lengths of Fe–Fe, As–Fe, and Li–Fe are 2.597 Å, 2.468 Å, and 2.911 Å, respectively. The corresponding bond angles Fe–Fe–Li and As–Li–Fe are 116.499$\phantom{0}^{\circ }$ and 104.855$\phantom{0}^{\circ }$, consistent with literature values^44^. These findings highlight the reliability of the present structural model and establish a solid basis for analyzing the modifications introduced by Ru doping in LiFeAs.

**Fig. 1:**
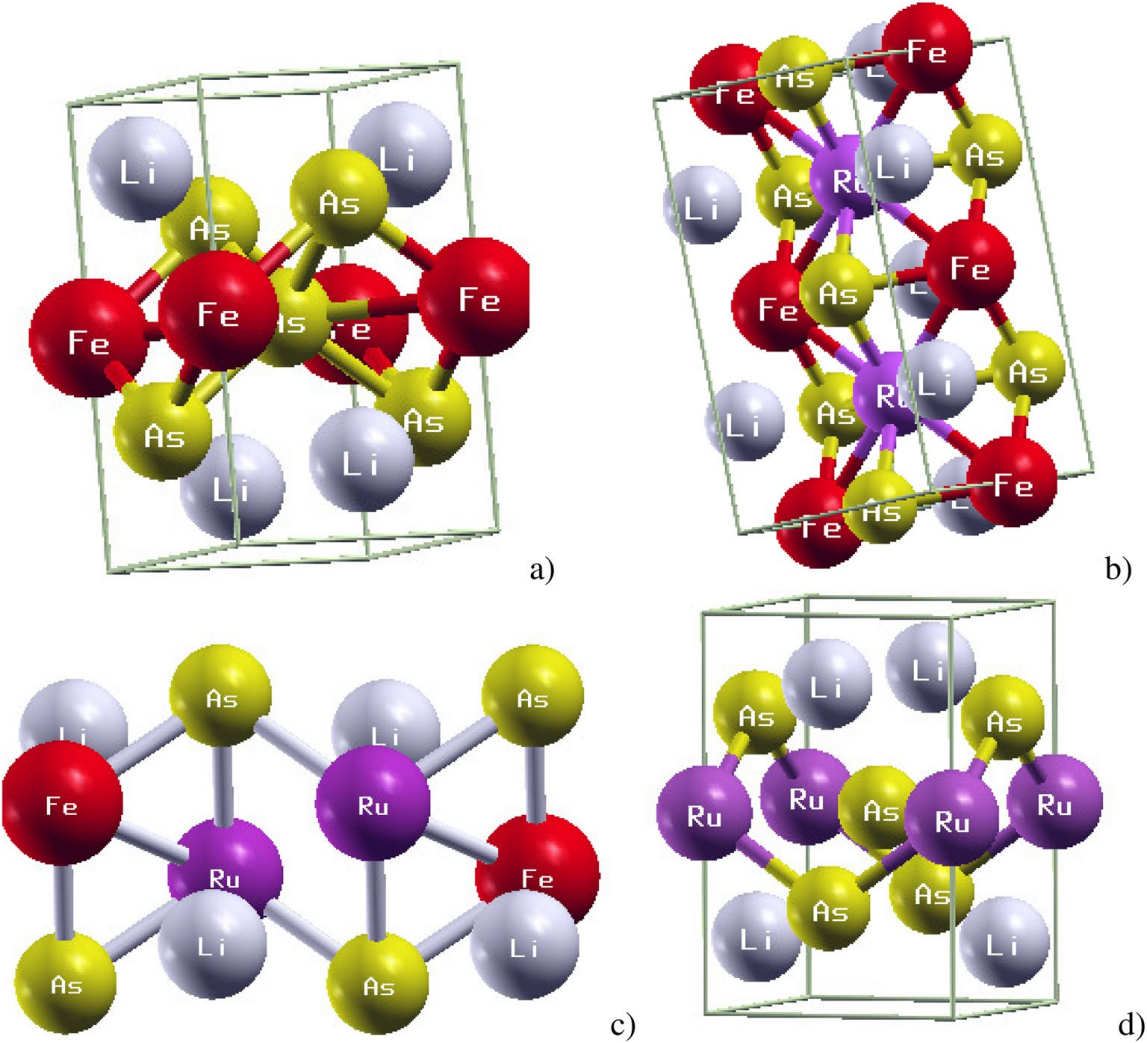
Crystal structure of pristine (**a**), 25% (**b**) 50% (**c**) and 100% (**d**) Ru-doped LiFeAs .

Figure 1 presents the optimized crystal structures of pristine (a), 25% (b), 50% (c), and 100% (d) Ru-substituted LiRuAs. In pristine, the FeAs layers are well preserved with Li ions intercalated between them, forming the characteristic layered architecture typical of iron-based superconductors. Upon 25% Ru substitution, modeled using a $2 \times 2 \times 1$ supercell, a modest lattice expansion is observed, reflecting the structural response to partial replacement of Fe by Ru. The use of a larger supercell at this doping level is essential to realistically capture substitutional disorder, though it results in Brillouin-zone folding and an apparent increase in the number of electronic bands. At 50% Ru doping, treated within a $2 \times 1 \times 1$ cell, the lattice undergoes further expansion, demonstrating the progressive influence of Ru incorporation while maintaining the layered framework. Finally, in the fully substituted LiRuAs phase, the lattice expands substantially compared to pristine , consistent with the larger ionic radius of Ru relative to Fe. Nonetheless, the optimized lattice parameter of 4.062Å remains smaller than the reported theoretical value of 5.60Å^45^.

**Table 1:** The optimized lattice parameters (calculated, experimental and theory) result of Ru-doped LiFeAs.

Technique	System	Calculated value	Literature: Experiment	Literature : Theory
DFT	LiFeAs	a = 3.767 Å, c = 6.230 Å	The lattice parameter of LiFeAs: a = 3.771 Å, c = 6.350 Å^46^	a = 3.769 Å, c = 6.307 Å ^49^
	LiFe$\phantom{0}_{0.75}$Ru$\phantom{0}_{0.25}$	a = 3.786 Å, c = 6.420 Å		
	LiFe$\phantom{0}_{0.5}$Ru$\phantom{0}_{0.5}$As	a = 3.896 Å, c = 6.430 Å		
	LiRuAs	a = 4.062 Å, c = 6.470 Å		The optimized lattice constant a=c=5.601 Å^45^
DFT+U	LiFeAs	a = 3.779 Å, c = 6.382 Å		
	LiFe$\phantom{0}_{0.5}$Ru$\phantom{0}_{0.5}$As	a = 4.023 Å, c = 6.455 Å		
	LiRuAs	a = 4.090 Å, c = 6.485 Å		

Table 1 indicates the influence of electron correlation effects on the structural properties of Ru-doped LiFeAs, comparing results obtained from standard DFT with those incorporating Hubbard corrections (DFT+*U*). Across all systems studied, the inclusion of *U* leads to a systematic increase in lattice parameters, with the magnitude of expansion quantified in terms of relative percentage change. For pristine , the lattice constants increase from a = 3.767 Å and c = 6.230 Å (DFT) to a = 3.779 Å and c = 6.382 Å (DFT+*U*). This corresponds to expansions of approximately 0.3% along the “a”-axis and 0.08% along the “c”-axis, indicating that electron correlation captured by the Hubbard *U* exerts a more pronounced influence on the interlayer separation (“c”-axis) than on the in plane lattice spacing. At 50% Ru substitution (LiFe$\phantom{0}_{0.5}$Ru$\phantom{0}_{0.5}$As), the lattice constants expand from a = 3.896 Å and c = 6.430 Å (DFT) to a = 4.023 Å and c = 6.455 Å (DFT+*U*), corresponding to relative increases of 0.27% and 0.35%, respectively. In the fully substituted LiRuAs compound, the expansion is more moderate, with the “a”-axis and “c”-axis increasing by 0.095% and 0.073% under DFT+*U*, respectively. These results highlight that Ru incorporation, owing to its more delocalized 4*d* orbitals, reduces the sensitivity of the lattice to correlation effects compared to Fe-rich compositions. Overall, the inclusion of Hubbard *U* consistently enhances the lattice parameters, with anisotropic expansion most evident along the “c”-axis. This trend is consistent with the enhanced localization of Fe 3*d* electrons, where electron correlation weakens bonding interactions and drives lattice expansion. Previous studies have demonstrated that DFT+*U* yields lattice parameters in close agreement with experiment for LiFeAs ^47^,and the use of first-principles *U* parameters ^48^ and orbital-resolved extensions of the *DFT+U * method further refine structural accuracy.

.

## Electronic band structure of Ru-doped LiFeAs

**Fig. 2:**
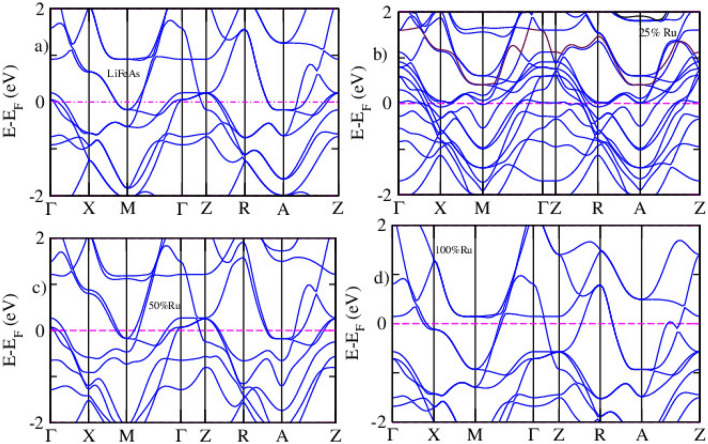
Calculated band structures of pristine (**a**), 25% (**b**), 50% (**c**), and 100% (**d**) Ru-doped LiFeAs obtained using DFT.

The band structure of LiFeAs is central to understanding its electronic behavior and the mechanisms governing superconductivity^51^. Substituting Ru at the Fe sites in LiFeAs, at concentrations of 25%, 50%, and 100%, introduces substantial modifications to the electronic structure. These changes arise primarily from the distinct electronic configuration of Ru, whose more delocalized 4*d* orbitals differ significantly from the relatively localized Fe 3*d* orbitals. As a result, Ru substitution alters orbital hybridization near the Fermi level, reshaping the DOS and modifying the band dispersion. The calculated band structures, plotted within an energy window from –2 to +2 eV relative to the Fermi level, clearly illustrate progressive band shifts and redistribution of electronic states with increasing Ru concentration (Fig. 2). For pristine , multiple Fe–3*d* derived bands cross the Fermi level, giving rise to electron and hole pockets that are widely recognized as essential features for superconductivity in Fe-based materials. With 25% Ru doping, the overall band topology is preserved, but a modest upward shift of the Fermi level ($E_F$) and band broadening are evident, reflecting both the presence of Ru’s additional valence electrons and enhanced hybridization effects. At higher doping levels more pronounced modifications appear. The bands become increasingly dispersive due to the greater delocalization of Ru-4*d* orbitals, while the Fermi level shifts further upward. Some bands near $E_F$ are pushed below the Fermi level, reducing the DOS at the Fermi energy. This reduction indicates a suppression of electronic correlations and magnetic fluctuations, which are widely considered critical to unconventional superconductivity in these systems. Consequently, while Ru doping enhances metallicity through broader band dispersion, it simultaneously weaken superconductivity by diminishing the electronic interactions necessary for Cooper pairing.

Figure 2 presents the electronic band structures of LiFe$\phantom{0}_{1-x}$Ru$\phantom{0}_{x}$As for x = 0.25, 0.50, and 1.00, obtained from DFT calculations. The pristine compound shows several Fe-3*d*–derived bands crossing the Fermi level ($E_F$), confirming its metallic character with well-defined electron and hole pockets, which are crucial for superconductivity. Upon 25% Ru substitution, the overall band topology remains largely preserved; however, a slight upward shift of $E_F$ and a moderate band broadening are evident. These changes are associated with the increased carrier concentration arising from the additional valence electron of Ru compared to Fe. At higher Ru concentrations ($x =$ 0.50 and 1.00), more pronounced modifications appear. The bands become increasingly dispersive, reflecting the more delocalized nature of Ru-4*d* orbitals relative to Fe-3*d*. The Fermi level shifts further upward, while some near-$E_F$ bands sink below it, leading to a reduction in the density of states at $E_F$. Such behavior indicates that Ru doping enhances metallicity but may simultaneously suppress superconductivity by diminishing electronic correlations and magnetic fluctuations.

**Fig. 3:**
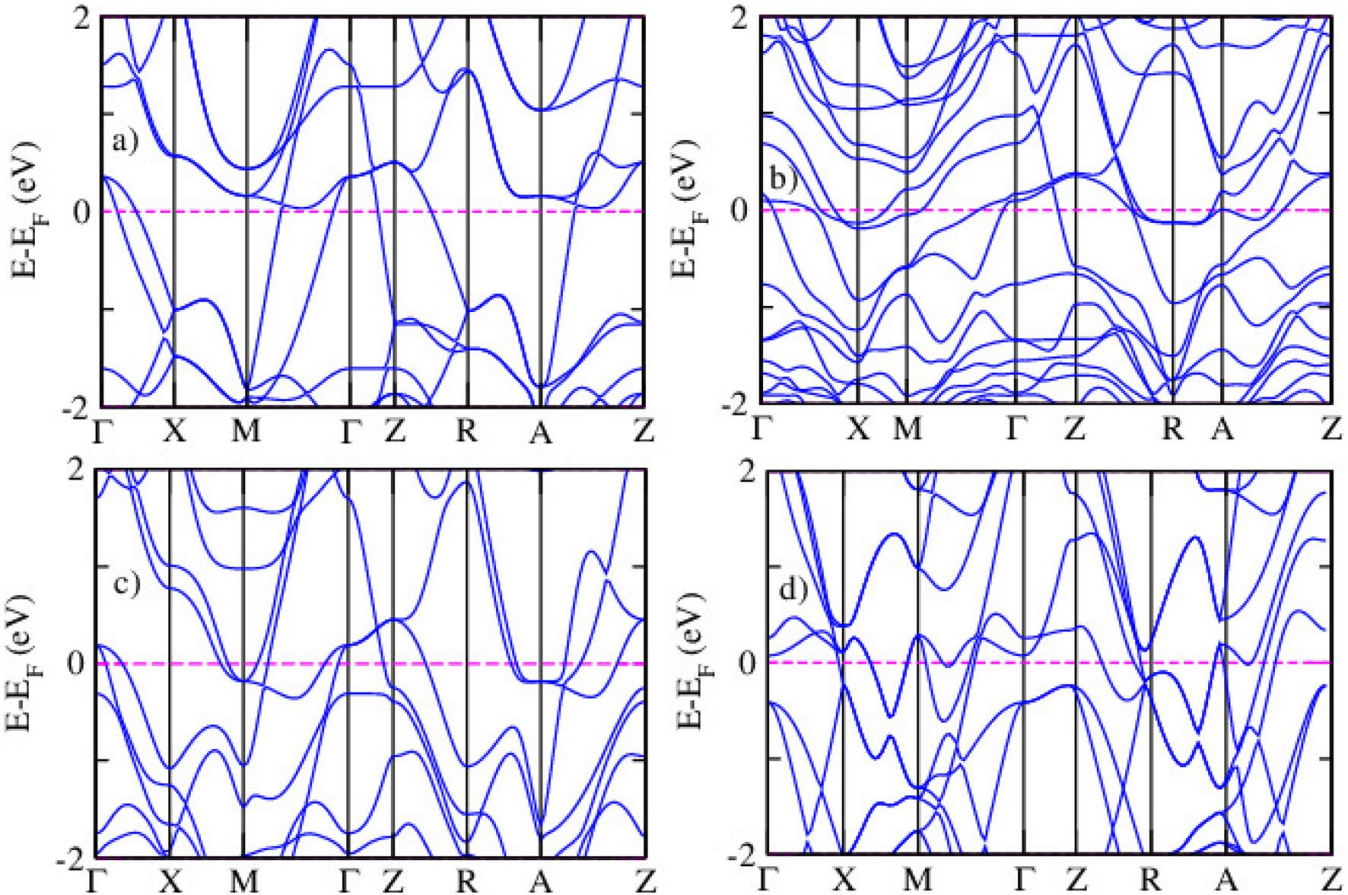
Band structure of pristine (**a**), 25% (**b**), 50% (**c**) and 100% (**d**) Ru-doped LiFeAs via DFT+*U* calculation.

Figure 3 shows the electronic band structures of pristine and Ru-doped LiFeAs compounds calculated using the DFT+*U* method. The subfigures include (a) pristine LiFeAs, (b) 25% , (c) 50% , and (d) fully substituted LiRuAs. In the pristine LiFeAs system (Fig. 3a), multiple bands cross the Fermi level ($E_\textrm{F}$), confirming its metallic nature. The bands near $E_\textrm{F}$ primarily originate from Fe 3*d* states hybridized with As 4*p* orbitals. Prominent hole-like bands at the $\Gamma$ point and electron-like bands near the *M* point are consistent with characteristic features of iron-based superconductors. Upon 25% Ru substitution, Fig. 3b, the overall metallic character is preserved, though moderate shifts in the bands are observed. Band flattening near $E_\textrm{F}$ becomes noticeable, along with slight band splitting, indicating the influence of Ru 4*d* states interacting with the Fe sublattice. These modifications may affect the density of states and the nesting conditions that play a role in superconducting pairing mechanisms. At 50% Ru substitution Fig. 3c, the band structure shows more significant alterations. There is a reduced number of bands crossing the Fermi level and a more pronounced flattening of the bands, especially near high-symmetry points like $\Gamma$ and *Z*. These features suggest enhanced electronic correlation and possible localization tendencies, which could influence magnetic fluctuations and superconductivity. For fully substituted LiRuAs (Fig. 3d), the band dispersion is further modified, with fewer bands near $E_\textrm{F}$ and a lower density of states. The metallic nature is maintained; however, the electronic structure differs substantially from the pristine phase, likely due to the broader and more delocalized Ru 4*d* orbitals. These changes imply that Ru substitution weakens electronic correlations and may suppress magnetic excitations crucial for superconductivity. The DFT+*U* approach is essential for capturing the effects of electron correlation, particularly in partially localized 3*d* and 4*d* systems.

**Fig. 4:**
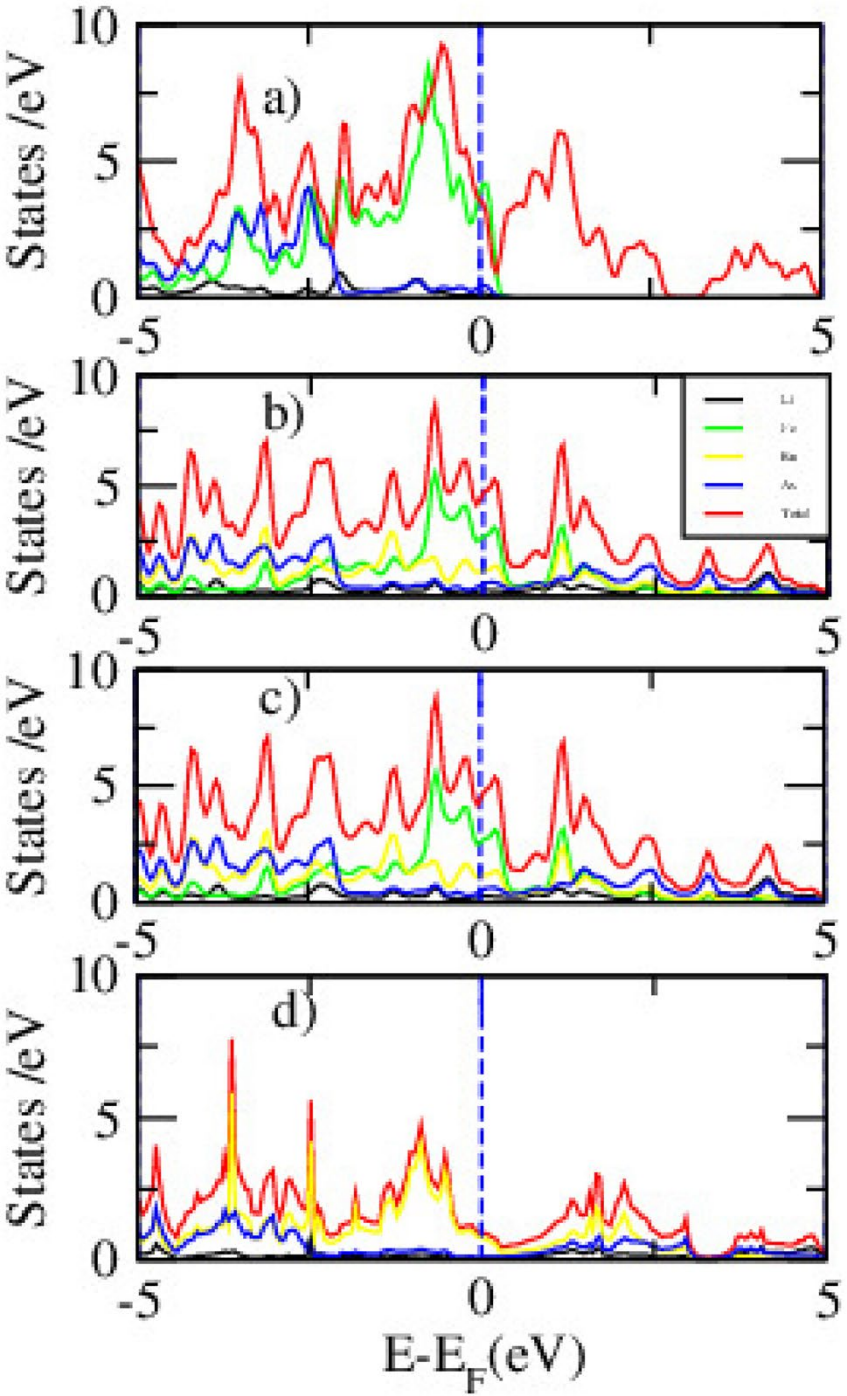
The partial density states of pristine (**a**), 25% (**b**), 50% (**c**), and 100% (**d**) Ru-doped LiFeAs via DFT calculation.

Figure 4 indicates the PDOS for pristine and Ru-doped LiFeAs with x = 0.00, 0.25, 0.50, and 1.00, obtained using DFT. The evolution of PDOS reveals how Ru doping influences the orbital contributions near the E$\phantom{0}_{F}$ and modifies the electronic structure. In the pristine compound (Fig. 4a), the Fe 3*d* orbitals dominate near E$\phantom{0}_{F}$, with significant hybridization from As 4*p* states. This confirms the multiband metallic nature of LiFeAs and its potential for superconductivity. The Li orbitals contribute minimally near E$\phantom{0}_{F}$. With 25% Ru doping Fig. 4b, Ru 4d states begin to emerge and overlap with Fe 3d orbitals, altering the density of states around the Fermi level. The Fe-3*d* peak becomes broader and less intense, indicating reduced electronic localization and a change in magnetic character. While the system remains metallic, a slight reduction in N(E$\phantom{0}_{F}$) is observed. At 50% Ru doping Fig. 4c, the Ru 4*d* contribution increases significantly, becoming comparable to Fe 3*d*. This results in a wider PDOS distribution and reduced peak sharpness near E$\phantom{0}_{F}$, suggesting greater band dispersion and delocalization. These changes may weaken magnetic interactions and alter the electronic correlation strength. In the fully substituted LiRuAs (Fig. 4d), Fe 3*d* states are completely replaced by Ru 4d orbitals. The PDOS near E$\phantom{0}_{F}$ becomes flatter and broader, reflecting highly delocalized electronic behavior. The N(E$\phantom{0}_{F}$) is further reduced, which may correlate with diminished magnetic exchange and potential suppression of superconductivity

**Fig. 5:**
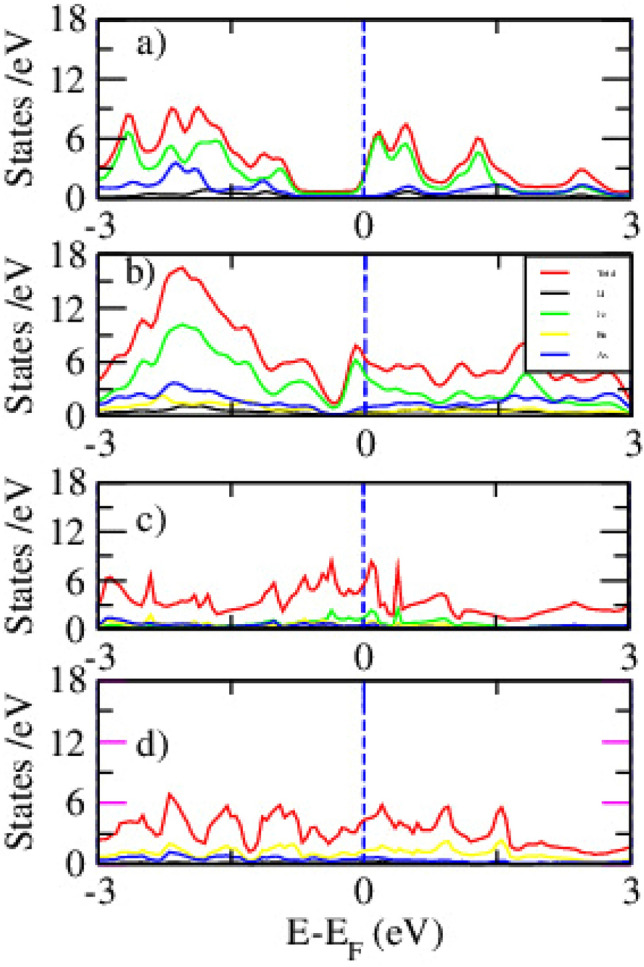
The partial density states of pristine (**a**), 25% (**b**), 50% (**c**), and 100% (**d**) Ru doped LiFeAs via DFT+*U* calculation.

Figure 5 displays the spin-polarized PDOS for LiFe$\phantom{0}_{1-x}$Ru$\phantom{0}_{x}$As at doping levels of x = 0.00, 0.25, 0.50, and 1.00, calculated using the DFT+*U* approach with U = 5.0 eV applied to Fe 3*d* orbitals. The results reveal how Ru substitution modifies the electronic structure and magnetic character of the system. In the pristine compound Fig. 5a, the Fe 3d orbitals dominate near the E$\phantom{0}_{F}$, exhibiting strong spin polarization. The hybridization between Fe 3*d* and As 4*p* states contributes to metallic behavior and magnetic interactions. The application of Hubbard *U* shifts part of the 3*d* states away from E$\phantom{0}_{F}$, enhancing localization and reflecting electron correlation. With 25% Ru substitution Fig. 5b, the Ru 4*d* states begin to appear near E$\phantom{0}_{F}$ and mix with Fe 3*d* states. This causes a reduction in the Fe 3*d* peak intensity and spin asymmetry, indicating partial suppression of magnetism. The system remains metallic, though N(E$\phantom{0}_{F}$) is slightly reduced. At 50% doping Fig. 5c, the Ru contribution becomes more pronounced, and Fe-related peaks are further suppressed. The PDOS shows broader, more delocalized states with weakened spin polarization, suggesting reduced magnetic ordering and electronic correlation. In the fully Ru-substituted LiRuAs Fig. 5d, the PDOS is dominated by Ru 4*d* orbitals, which are more delocalized and display minimal spin splitting.

**Table 2:** The Fermi energy and Binding energy of LiFe$\phantom{0}_{(1-x)}$Ru$\phantom{0}_{x}$As , (x= 0.25, 0.50,1.00) via DFT.

x	E$\phantom{0}_{F}$ (eV)	Binding energy (eV)	Binding energy/atom (eV)
0.00	9.35	−33.74	−5.58
0.25	9.56	−34.28	−5.85
0.50	9.79	−35.80	−5.98
1.00	10.03	−37.96	−6.26

Table  2 indicates the calculated Fermi energies and binding energies of LiFe$\phantom{0}_{1x}$Ru$\phantom{0}_{x}$As for Ru doping levels of x = 0.00, 0.25, 0.50, and 1.00 using standard DFT calculations. The results provide insight into the effect of Ru substitution on the electronic structure and thermodynamic stability of the system. The E$\phantom{0}_{F}$ shows a clear increasing trend with increasing Ru content. Specifically, E$\phantom{0}_{F}$ increases from 9.35 eV for pristine (x = 0.00) to 10.03 eV at full substitution (x = 1.00) an overall increase of approximately 7.3%. This upward shift reflects a systematic modification of the electronic structure caused by Ru doping, due to the more delocalized nature of Ru 4*d* orbitals compared to Fe 3*d*. These orbitals introduce additional electronic states near the Fermi level, effectively shifting E$\phantom{0}_{F}$ upward and indicating enhanced electronic activity as doping increases. The binding energy (BE), which represents the total energy gained when the compound forms from isolated atoms, becomes more negative with higher Ru substitution, indicating increasing structural stability. For the undoped system (x = 0.00), the total BE is 33.74 eV, while for x = 1.00 it reaches 37.96 eV, corresponding to a 12.5% increase in binding strength. This trend suggests that Ru incorporation strengthens the cohesive energy of the crystal, likely due to enhanced hybridization between Ru 4*d* and As 4*p* orbitals.

A more direct comparison is offered by the binding energy per atom, which also becomes more negative with doping: from 5.58 eV at x = 0.00 to 6.26 eV at x = 1.00, representing a  12.2% improvement in average atomic stability. The most significant gain occurs between 25% and 50% doping, where the BE/atom increases by about 2.2%, suggesting an energetically favorable interaction between Ru and the Fe–As lattice network. These results indicate that moderate to high Ru substitution not only modifies the electronic density of states near the Fermi level but also improves the thermodynamic stability of the host system. The consistent increase in both E$\phantom{0}_{F}$ and BE supports the notion that Ru doping stabilizes the LiFeAs structure while enhancing its electronic character, potentially influencing magnetic ordering and superconducting properties^52^. The DFT+*U* results indicate a gradual increase in Fermi energy and a slight change in binding energy per atom across the doping levels summarized in Table 3.

**Table 3:** The Fermi energy and Binding energy of LiFe$\phantom{0}_{(1-x)}$Ru$\phantom{0}_{x}$As (x = 0.25, 0.50, 1.00) via DFT+U calculation.

x	E$\phantom{0}_{F}$ (eV)	Binding energy (eV)	Binding energy/atom (eV)
0.00	9.0779	−38.23	−6.53
0.25	9.8064	−38.64	−6.39
0.50	10.4698	−39.32	−6.53
1.00	10.7524	−40.68	−6.80

The Fermi energy increases progressively with Ru content, rising from 9.08 eV at 0% Ru to 10.75 eV at 100% Ru. This corresponds to an overall increase of approximately 18.4%, reflecting the enhanced electronic contribution from Ru 4*d* states near the Fermi level. In terms of total binding energy, the system becomes more stable as Ru concentration increases. The binding energy improves from 38.23 eV (0% Ru) to 40.68 eV (100% Ru), representing an overall increase of about 6.4% in total stability. However, the binding energy per atom changes more subtly: from 6.53 eV at 0% Ru to 6.80 eV at 100% Ru, indicating a  4.1% increase in atomic bonding strength at full substitution. Interestingly, at 50% Ru doping, the per-atom binding energy remains equal to that of the pristine system (6.53 eV), suggesting that up to 50% substitution retains the original structural stability. At 25% Ru, the binding energy per atom slightly decreases to 6.39 eV (2.1% reduction), possibly due to local structural distortions. The most negative binding energy per atom at 100% Ru (6.80 eV) implies a stronger average bonding, likely influenced by Ru–As hybridization. These percentages confirm that Ru doping enhances both the electronic activity and thermodynamic stability of LiFeAs, particularly at higher doping levels^53^.

### Magnetic electronic structure

**Fig. 6:**
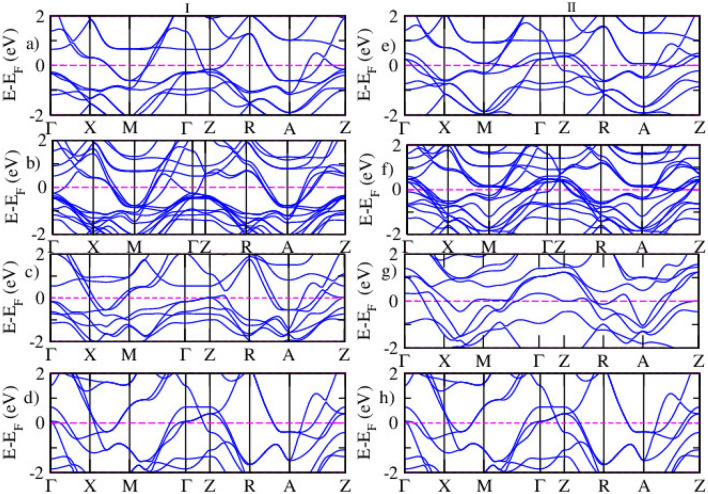
The spin-polarized electronic band structure for spin-up (I) and spin-down (II) states of pristine (**a**,**e**), 25% (**b**,**f**), 50% (**c**,**g**), and 100% (**d**,**h**) Ru-doped LiFeAs, as calculated via DFT calculation.

The magnetic properties are analyzed by examining the total energy difference between ferromagnetic and antiferromagnetic spin-polarized calculations, along with the magnetic moments of the systems^54^. In a ferromagnetic material, the absolute magnetization is equal to the total magnetization, while in an antiferromagnetic system, the total magnetization is zero, and the absolute magnetization is twice the magnetization of each of the two atoms in the system. This behavior aligns with the magnetic models used in this study. The experimental magnetic moment of iron in LiFeAs is reported to be 3.4$\mu _B$^55^. Previous theoretical studies have shown that the calculated magnetic moments for iron in similar systems tend to be lower, often due to challenges in accurately capturing strong electron correlations and structural effects in computational models. The magnetic moment of the Ru atom is 2.35 $\mu _B$. The spin-polarized electronic band structures of LiFe$\phantom{0}_{1-x}$Ru$\phantom{0}_{x}$As (x = 0.25, 0.5, 1) are calculated for both magnetic configurations using DFT approximations, as shown in Fig. 6. The DFT results confirm that the LiFe$\phantom{0}_{1-x}$Ru$\phantom{0}_{x}$As systems remain metallic.

Figure 6 shows the band structures of LiFe$\phantom{0}_{(1-x)}$Ru$\phantom{0}_{x}$As (x = 0.25, 0.5, 1), demonstrating that Ru substitution progressively reduces the metallic nature of the material. As the Ru concentration increases to 25% and 50%, a noticeable decrease in the number of bands at the Fermi level is observed, signifying a reduction in metallicity and suggesting a weakening of superconductivity. At full Ru substitution, the material approaches a nonmetallic state, characterized by a substantial decrease in bands at the Fermi level and a lowered density of states, making superconductivity highly unlikely. These observations indicate that increasing Ru concentration suppresses both the metallic and superconducting properties of LiFeAs. These findings are in agreement with Singh’s work^56^, which examined the doping effects in iron-based pnictides BaFe$\phantom{0}_{2}$As$\phantom{0}_{2}$ and LiFeAs, showing that doping reduces the degree of Fermi surface nesting, thereby diminishing metallicity and impacting superconductivity. Additionally^57^ reported similar trends in Sm(Fe$\phantom{0}_{1-x}$Ru$\phantom{0}_{x}$)As($\phantom{0}_{0.85}$F$\phantom{0}_{0.15}$), where Ru doping (for x $\ge$ 0.5) led to a suppression of superconductivity and a transition to a metallic state. Figure 6 shows the spin-polarized band structure of the FM configuration. For the AFM case, the spin-up and spin-down bands are identical due to the symmetry of the AFM ordering; thus, only the FM band structure is shown here to highlight the spin-splitting behavior.

**Fig. 7:**
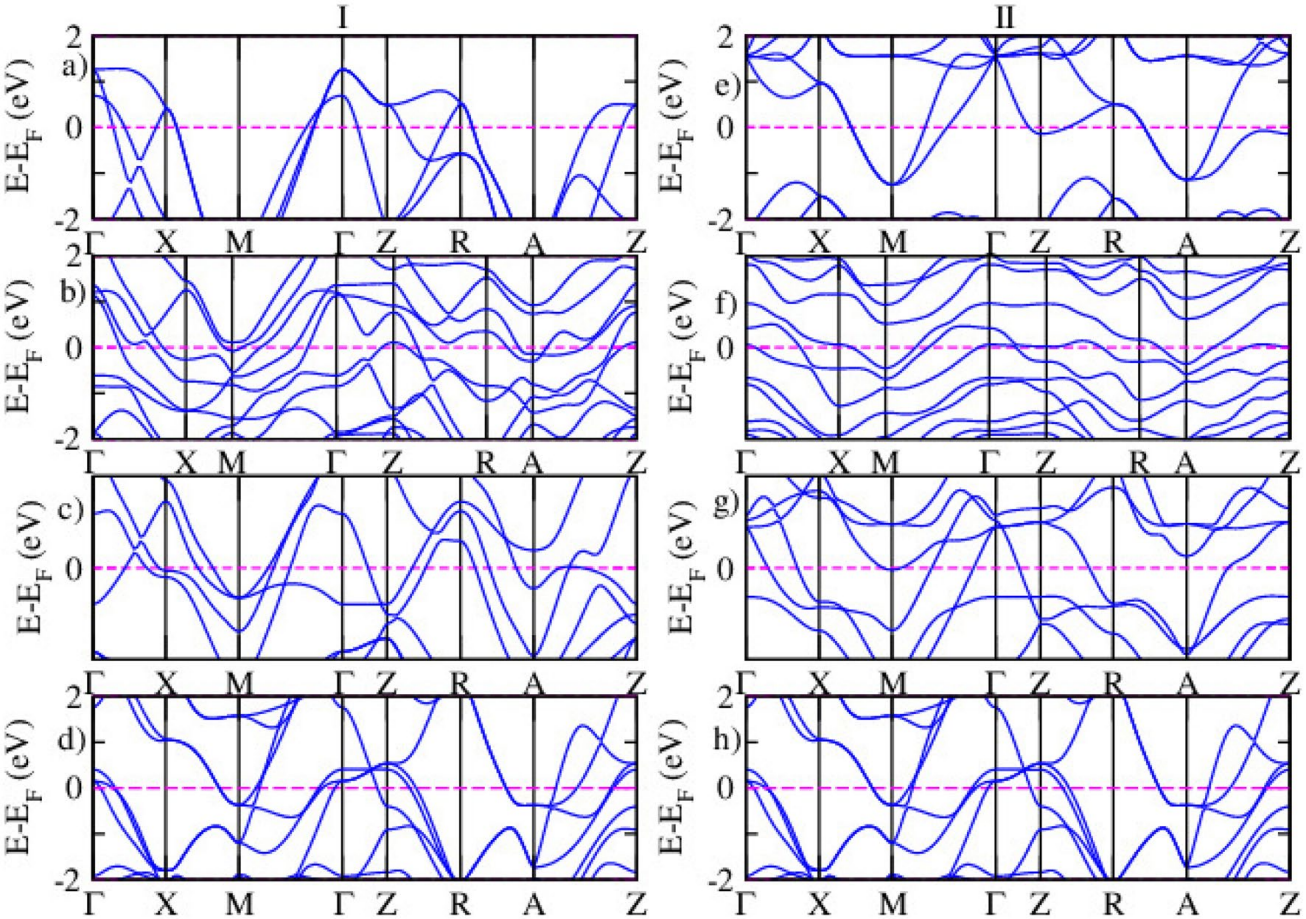
The spin polarized electronic band structure for spin up (I) and spin down (II) states of pristine (**a**,**e**), 25% (**b**,**f**), 50% (**c**,**g**), and 100% (**d**,**h**) Ru doped LiFeAs via DFT+*U* calculation.

Figure 7 indicates the spin-polarized electronic band structures of pristine and Ru-doped LiFeAs systems calculated using the DFT+*U* approach. In the pristine compound Fig. 7a,e, both spin-up and spin-down band structures show nearly symmetrical behavior, consistent with a weak or absent magnetic moment, characteristic of the non-magnetic or slightly magnetic ground state of LiFeAs. As Ru is gradually introduced into the lattice, noticeable spin asymmetry begins to emerge. In the 25% Ru-doped structure Fig. 7b,f, slight spin splitting appears near the Fermi level, indicating the onset of spin polarization and the influence of Ru 4*d* orbitals on the electronic structure. At 50% Ru substitution, Fig. 7c,g, the spin asymmetry becomes more pronounced, particularly in the vicinity of the Fermi level along the high-symmetry directions. The band dispersion shows flatter bands in some regions, which may suggest increased electron correlation and partial localization of Fe-3*d* and Ru-4*d* states. Importantly, a reduced band crossing at the Fermi level in one spin channel hints at a potential imbalance in the density of states between spin-up and spin-down electrons, which may have implications for magnetic ordering and spin transport. In the fully substituted LiRuAs system Fig. 7d,h, a more distinct spin polarization is observed, with a clear difference between spin-up and spin-down bands across the Brillouin zone. The conduction and valence band edges show notable separation between the spin channels, suggesting that Ru substitution enhances magnetic effects and could induce a ferromagnetic-like ground state, depending on the exact arrangement of atoms and electron occupancy.

**Table 4:** The Fermi energy and binding energy of LiFe$\phantom{0}_{(1-x)}$Ru$\phantom{0}_{x}$As.

Magnetic order	x	E$\phantom{0}_{F}$ (eV)	Binding energy (eV)	Binding energy/atom (eV)
FM	0.00	9.49	−33.74	−5.62
	0.25	9.88	−34.01	−5.66
	0.50	10.14	−35.80	−5.97
	1.00	12.31	−36.87	−6.14
AFM	0.00	9.22	−33.87	−5.64
	0.25	10.13	−34.42	−5.73
	0.50	10.78	−35.78	−5.96
	1.00	12.31	−36.87	−6.14

Table 4 presents the E$\phantom{0}_{F}$, total binding energy, and binding energy per atom for LiFe$\phantom{0}_{1x}$Ru$\phantom{0}_{x}$As with varying Ru concentrations (x = 0.00, 0.25, 0.50, and 1.00), evaluated under both FM and AFM configurations using DFT calculations.

The E$\phantom{0}_{F}$ shows a consistent increase with rising Ru content for both FM and AFM magnetic states. For the FM case, E$\phantom{0}_{F}$ rises from 9.49 eV at x = 0.00 to 12.31 eV at x = 1.00, which is an approximate 29.7% increase, indicating significant modifications in the electronic structure due to the extended Ru 4*d* orbitals contributing more electronic states at the Fermi level. A similar trend is observed in the AFM configuration, where E$\phantom{0}_{F}$ increases from 9.22 to 12.31 eV, highlighting that Ru doping systematically alters the density of states near E$\phantom{0}_{F}$ regardless of spin ordering. The binding energy also reflects increased thermodynamic stability with doping. In the FM phase, the total binding energy increases from 33.74 eV (x = 0.00) to 36.87 eV (x = 1.00), amounting to a  9.3% gain in total cohesion energy. The per-atom binding energy follows a steady progression from 5.62 to 6.14 eV, showing a  9.9% improvement. These trends suggest that Ru incorporation enhances lattice stability by forming stronger Ru–As and Fe–As bonds, especially at higher doping levels.

In the AFM configuration, the same energetic pattern is observed: total binding energy increases from 33.87 to 36.87eV, and binding energy per atom improves from 5.64 to 6.14 eV as x goes from 0.00 to 1.00. Notably, the binding energy values for AFM are slightly more negative than their FM counterparts at each doping level, particularly at x = 0.25 and x = 0.50. This indicates a marginal energetic preference for AFM ordering in the intermediate doping range, suggesting enhanced magnetic exchange stabilization from Ru substitution at these concentrations. The consistent rise in E$$\phantom{0}_{F}$$ and binding energy with increasing Ru content confirms that Ru plays a stabilizing electronic and magnetic role in the system, potentially influencing its superconducting and magnetic behavior. The energy change $$\Delta$$E$$\phantom{0}_{Total}$$of the magnetic system is defined as the energy difference between the FM and AFM states of LiFeAs, expressed as follows: $$\Delta$$E$$\phantom{0}_{Total}$$ = E$$\phantom{0}_{Total}$$(FM)-E$$\phantom{0}_{Total}$$(AM) where, E$$\phantom{0}_{Total}$$(FM),  (E$$\phantom{0}_{Total}$$(AFM) ) is the total energy of the FM(AFM) ordering. The thermal energy equation for the system is expressed as $\Delta$ E/N=3/2K$_B$T[61], Where $\Delta$E is the total thermal energy of the system, N is the number of Fe per atom, K$_B$ is the Boltzmann constant and T is the Ne’el transition temperatutre of the AFM system. The magnetic energy difference of the Ru-substituted LiFeAs system confirms that the FM and AFM properties are tunable depending on the concentration and configuration of the system. LiFeAs are unconventional iron-based superconductors distinguished by their unique electronic structure and superconducting properties. The specific heat and energy changes associated with its superconducting state can be analyzed via the Bardeen-Cooper-Schrieffer (BCS) theory^58^. This theory, which describes conventional superconductors, provides a framework for understanding the relationships among the superconducting gap, critical temperature, and thermodynamic properties. However, the superconductivity in LiFeAs deviates from the predictions of BCS theory owing to its multiband nature and strong electron correlations, requiring additional theoretical approaches to fully capture its behavior^59^.

**Table 5:** The energy change of magnetic moments of AFM and NM, $\Delta$E(eV), No. Fe per atom(N) *Coupling*, T$\phantom{0}^{(MFA)}$ and T$\phantom{0}^{(corr)}$ of Ru-doped LiFeAs via DFT.

System	$\Delta$E(eV)	N	Coupling	T$\phantom{0}^{(MFA)}$	T$\phantom{0}^{(corr)}$
LiFe$\phantom{0}_{0.5}$Ru$\phantom{0}_{0.5}$As	0.0103	3	AFM	80.15	53.43
LiFe$\phantom{0}_{0.75}$Ru$\phantom{0}_{0.25}$As	0.0061	6	AFM	47.19	31.46
LiRuAs	0.000	0	NM	0.000	0.000

Table 5, the effects of Ru substitution on the magnetic coupling, energy change ($\Delta$E), and FM/AFM transition temperatures (T$\phantom{0}^{(MFA)}$ & T$\phantom{0}^{(corr)}$) for Ru doping increase, the energy change decreases, reflecting a weakening of AFM coupling. For LiFe$\phantom{0}_{0.5}$Ru$\phantom{0}_{0.5}$As and LiFe$\phantom{0}_{0.75}$Ru$\phantom{0}_{0.25}$As, the reduced $\Delta$E values (0.013eV and 0.006eV) lead to lower T$\phantom{0}^{(MFA)}$ (80.15 K and 47.19 K) and T$\phantom{0}^{(corr)}$ (53.43 K and 31.46 K). In fully substituted LiRuAs, the magnetic coupling transitions to nonmagnetic coupling, with no transition tempertautre (T=0).$\phantom{0}_{c}$ These results demonstrate that Ru substitution systematically suppresses magnetic interactions and superconductivity, transitioning the material from a strongly AFM coupled superconductor to a non-magnetic state. These findings are consistent with studies on other iron-based superconductors. In BaFe$\phantom{0}_{2}$As$\phantom{0}_{2}$, Ru substitution has been shown to significantly reduce superconductivity, with the suppression of AFM coupling becoming evident as the Ru concentration increases reported that for BaFe$\phantom{0}_{2-x}$Ru$\phantom{0}_{x}$As$\phantom{0}_{2}$, increasing Ru content reduced both the superconducting Néel transition temperature and the AFM ordering temperature, transitioning the material toward a nonmagnetic state with full substitution. Similarly reported^60^ that FeSe$\phantom{0}_{1-x}$Ru$\phantom{0}_{x}$ leads to the suppression of superconductivity, with the magnetic properties weakening as the Ru concentration increases.

**Table 6:** The energy change of magnetic moments of AFM and NM , $\Delta$E(eV), No. Fe per atom, *Coupling*, T$\phantom{0}^{(MFA)}$ and T$\phantom{0}^{(corr)}$ of Ru doped LiFeAs via DFT+*U*.

System	$\Delta$E(eV)	N	Coupling	T$\phantom{0}^{(MFA)}$	T$\phantom{0}^{(corr)}$
LiFe$\phantom{0}_{0.5}$Ru$\phantom{0}_{0.5}$As	0.0420	3	AFM	325.3	195.2
LiFe$\phantom{0}_{0.75}$Ru$\phantom{0}_{0.25}$As	0.0227	6	AFM	175.9	105.5
LiRuAs	0.0000	0	NM	0.000	0.000

Table6 indicates the magnetic energy differences $$\Delta$$E, magnetic coupling nature, and estimated Néel transition temperatures (T$$\phantom{0}_{c}$$) of Ru-doped LiFeAs systems computed via DFT+*U*. The energy difference $$\Delta$$E is defined as the total energy difference between the antiferromagnetic and ferromagnetic configurations $$\Delta$$E = E$$\phantom{0}_{FM}$$− E$$\phantom{0}_{AFM}$$, serving as an indicator of magnetic stability and ordering tendencies. For LiFe$$\phantom{0}_{0.5}$$Ru$$\phantom{0}_{0.5}$$As, a significant $$\Delta$$E of 0.0421 eV is observed, indicating strong AFM coupling and robust magnetic interactions. This corresponds to a high mean-field approximation (T$$\phantom{0}^{(MFA)}$$) = 325.3 K, with a corrected Néel transition temperature (T$$\phantom{0}^{(corr)}$$) = 195.2 K, suggesting magnetic ordering may persist well above room temperature within this composition. At 25% Ru doping, the AFM character remains, though $$\Delta$$E decreases to 0.0227 eV, leading to lower Néel transition temperatures ($$T^{(MFA)}$$ = 175.9 K, $$T^{(corr)}$$ = 105.5 K). This reduction reflects the weakening of AFM exchange interactions as Ru partially replaces Fe and disrupts the magnetic exchange pathways, due to the more delocalized nature of Ru’s 4d electrons. In contrast, the fully substituted LiRuAs system exhibits non-magnetic behavior with $$\Delta$$E = 0, and both $$T^{(MFA)}$$ and $$T^{(corr)}$$ are zero, confirming the complete suppression of magnetic ordering. This transition from an AFM ground state in Fe-rich compositions to a non-magnetic state in Ru-rich ones highlights the delicate balance between electron correlation and itinerancy introduced by Ru doping. $$\Delta$$$$\phantom{0}_B$$$$\phantom{0}_{c}$$ $$\Delta$$$$\phantom{0}_B$$$$\times 10^{-5}$$$$\phantom{0}_{c}$$.

### Magnetic density of states

A comprehensive understanding of the magnetic properties of Fe-based superconductors is crucial for elucidating the delicate interplay between magnetism and superconductivity. The magnetic DOS, particularly the spin-resolved PDOS, provides valuable insight into spin polarization effects and the evolution of magnetic ordering within these materials. In this work, spin-resolved PDOS calculations were performed for pristine and Ru-substituted counterparts using both DFT and DFT+*U* approaches, the latter employed to better account for on-site Coulomb interactions. The substitution of Fe with Ru introduces more delocalized 4*d* electrons, characterized by broader orbital distributions and weaker electronic correlations compared to the localized 3*d* states of Fe. This modification is expected to have a pronounced impact on both the electronic structure and the magnetic response of the system.

**Fig. 8:**
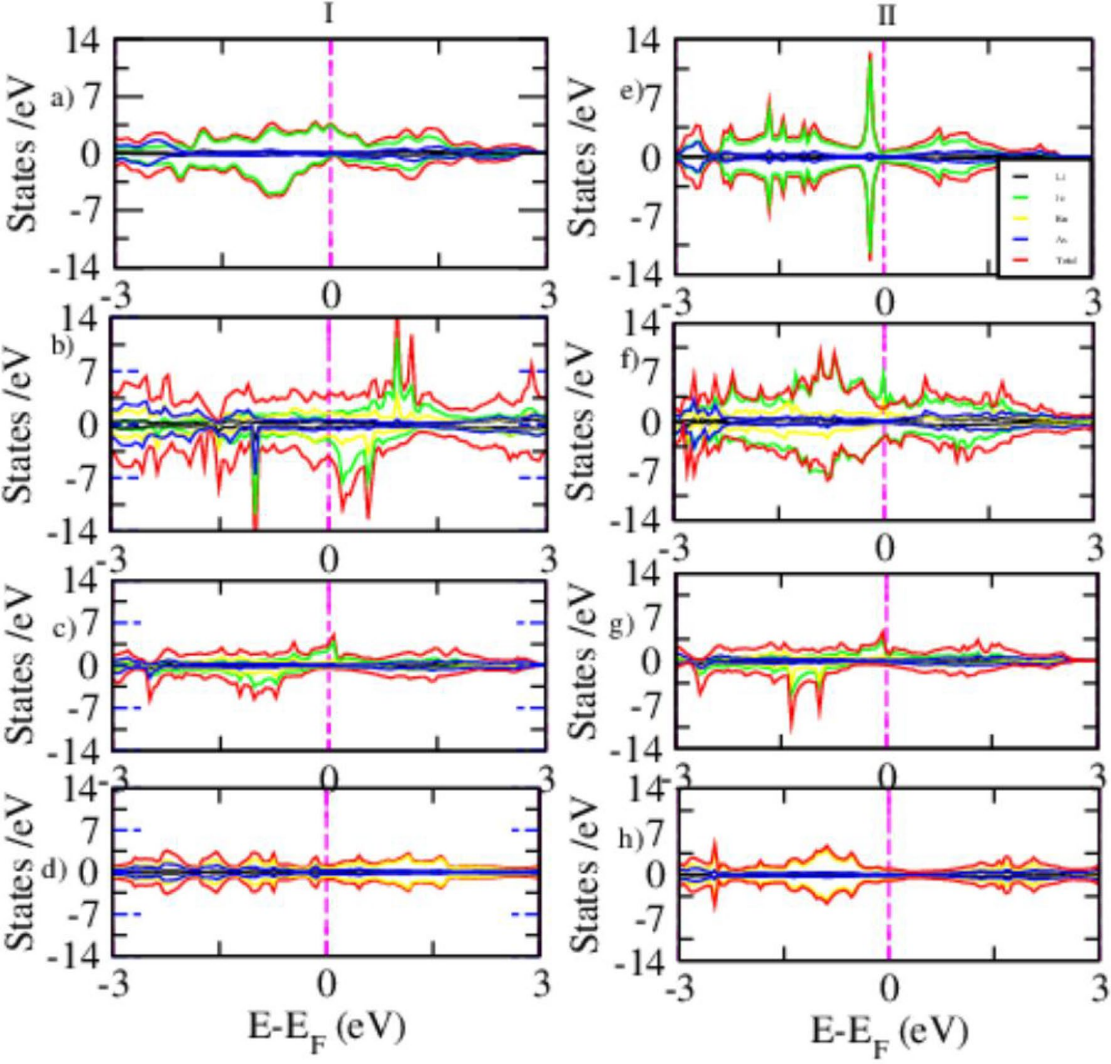
Spin-polarized PDOS for FM (I) and AFM (II) states of pristine (**a**,**e**), 25% Ru-doped (**b**,**f**), 50% Ru-doped (**c**,**g**), and 100% Ru-doped (**d**,**h**) systems, as obtained from DFT calculations.

Figure 8 shows the spin-polarized PDOS for both FM, (I) and AFM, (II) configurations of pristine and Ru-doped LiFeAs at substitution levels of 25%, 50%, and 100%. In pristine LiFeAs Fig. 8a,e, the FM state exhibits a clear asymmetry between spin-up and spin-down channels near the Fermi level, indicating spin polarization consistent with ferromagnetic ordering. By contrast, the AFM state shows nearly symmetric spin-up and spin-down DOS, consistent with antiferromagnetic alignment. Upon 25% Ru substitution Fig. 8b,f, both FM and AFM configurations begin to display enhanced spin asymmetry near the Fermi level, signaling the onset of Ru-induced spin polarization. The AFM state exhibits a stronger suppression of DOS at $E_F$, suggesting enhanced magnetic correlations relative to the FM configuration. At 50% doping Fig. 8c,g, the contrast between FM and AFM states becomes more pronounced: the AFM configuration shows sharper spectral features and reduced DOS at $E_F$, pointing toward localized magnetic ordering and possible frustration in the mixed Fe/Ru lattice. In the fully substituted LiRuAs compound Fig. 8d,h, the PDOS is dominated by Ru-4*d* states. The FM configuration shows broadened peaks and enhanced metallicity, while the AFM state maintains partial spin symmetry with weaker suppression at $E_F$. These results indicate that Ru substitution progressively destabilizes the delicate balance of magnetism in pristine LiFeAs and promotes complex spin fluctuations. Overall, the AFM configuration consistently exhibits lower DOS at the Fermi level across doping levels, suggesting it is energetically more favorable. This trend is consistent with previous findings in FeTe$\phantom{0}_{1-x}$Ru$\phantom{0}_{x}$, where high Ru content suppresses metallicity and superconductivity due to a vanishing DOS at $E_F$ in both FM and AFM configurations^62^.

**Fig. 9:**
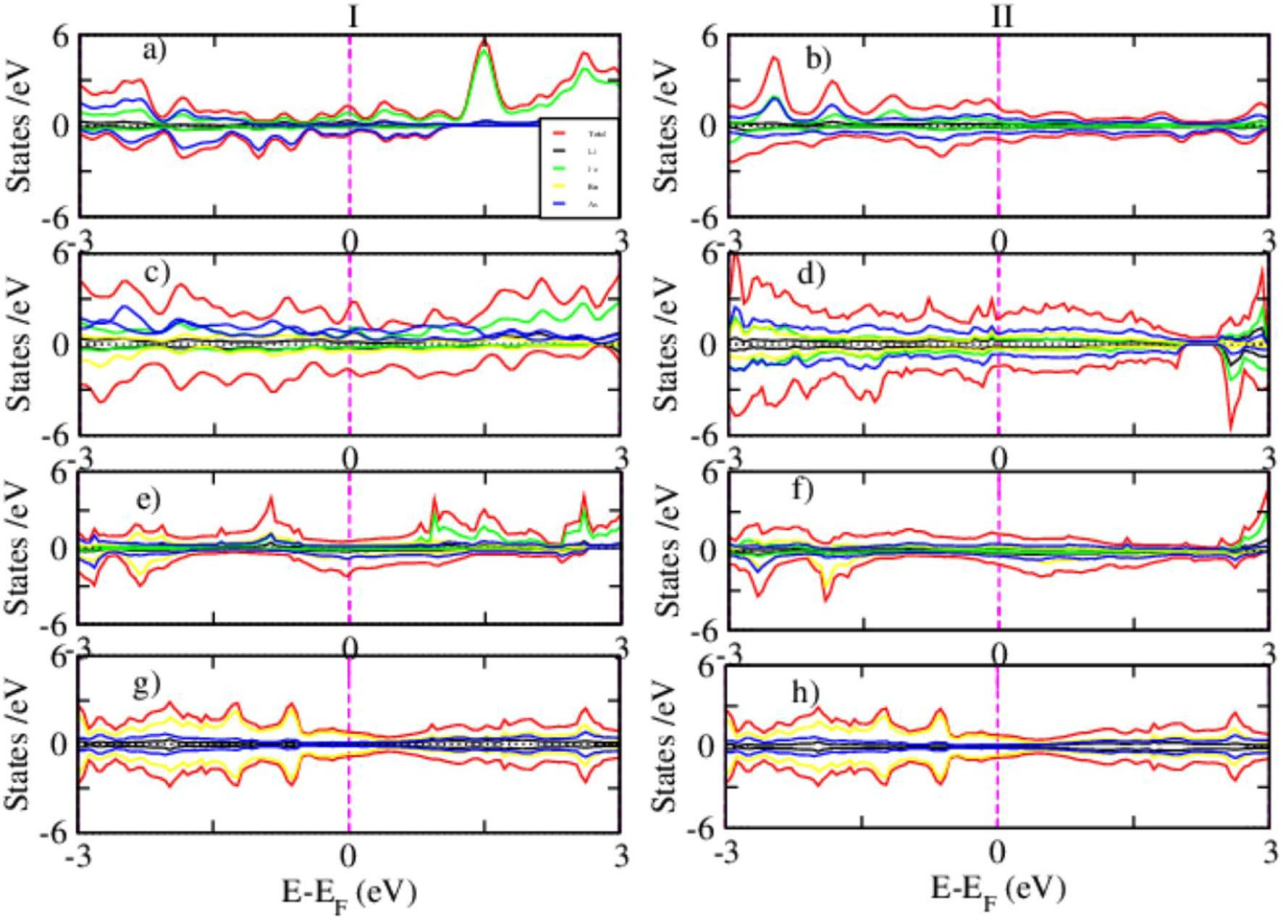
The PDOS for FM (I) and AFM (II) configurations of pristine (**a**,**b**), 25% (**c**,**d**), 50% (**e**,**f**), and 100% (**g**,**h**) Ru doped LiFeAs via DFT+*U* calculation.

Figure 9 presents the spin-polarized PDOS for FM (I) and AFM (II) configurations of pristine and Ru-doped LiFeAs, calculated using the DFT+*U* method. The inclusion of on-site Coulomb interactions highlights the role of electronic correlation in shaping the magnetic and electronic structure across different Ru concentrations. For pristine Fig. 9a,b, both FM and AFM states exhibit nearly antisymmetric spin-up and spin-down PDOS, with dominant contributions from Fe-3*d* orbitals near the Fermi level. Compared to standard DFT, the DFT+*U* approach narrows the bandwidth and enhances Fe-3d localization, consistent with stronger electron correlation effects. The AFM configuration shows a slight suppression of DOS at $E_F$, suggesting a marginal energetic preference and aligning with the proximity to magnetic ordering reported experimentally^63^. Upon 25% Ru substitution Fig. 9c,d, hybridization between Fe-3*d* and Ru-4*d* orbitals becomes evident, accompanied by stronger spin asymmetry in the AFM state. The reduction of DOS at $E_F$ in the AFM configuration indicates enhanced magnetic stability, consistent with Ru’s disruption of itinerant magnetism. At 50% Ru doping Fig. 9e,f, Fe-derived states are further suppressed and Ru-4*d* contributions dominate. The AFM PDOS develops sharper features and a partial gap near $E_F$, suggesting reduced metallicity and a possible tendency toward localized magnetic ordering or weakened superconductivity. At full Ru substitution Fig. 9g,h, the system becomes Ru-4*d* dominated. Both FM and AFM states remain metallic, though the FM configuration exhibits a higher DOS at $E_F$, implying a relative stabilization of FM tendencies. Across the series, AFM configurations generally display reduced DOS at $E_F$, highlighting their greater relative stability and underscoring the delicate balance between electronic correlation, magnetism, and superconductivity in these systems.

J$\phantom{0}_{1}$ =E $\phantom{0}_{AFM1}$ − E$\phantom{0}_{FM}$/8, J$\phantom{0}_{2}$ = E$\phantom{0}_{AFM2}$ − E$\phantom{0}_{FM}$ − 4J$\phantom{0}_{1}$/8. The enhanced J$\phantom{0}_{1}$ and variable J$\phantom{0}_{2}$ trends provide further insight into the suppression or stabilization of magnetic ordering with doping. This model assumes a square Fe sublattice with spin $S=1$. The calculated $J_1$ and $J_2$ values for 25% and 50% Ru-doped LiFeAs are: DFT: 25% Ru: $J_1 = 0.76$ meV, $J_2 = -1.42$ meV; 50% Ru: $J_1 = 1.28$ meV, $J_2 = -0.22$ meV. DFT+*U*: 25% Ru: $J_1 = 2.84$ meV, $J_2 = 0.14$ meV; 50% Ru: $J_1 = 5.25$ meV, $J_2 = -0.21$ meV. These results clearly indicate that Ru doping has a profound impact on the magnetic exchange interactions. At 25% substitution, the positive values of $J_{1}$ and $J_{2}$ promote AFM coupling, reflecting a tendency toward stable AFM order. However, at 50% doping, both the reduction in magnitude and the sign reversal of the exchange parameters signal the weakening of long-range magnetic order and the emergence of pronounced magnetic frustration and instability an effect that becomes particularly evident within the DFT+*U* framework.

### Lattice dynamics in LiFe_1−x_Ru_x_As material

Phonon dispersion plays a crucial role in iron-based superconductors (IBSCs), as it provides key insights into the interaction between lattice vibrations and the electronic system^64^. This coupling is particularly important in compounds like LiFeAs, where elemental doping, such as with Ru, can alter both the phonon spectrum and the superconducting behavior. The phonon dispersion curves for both pristine and Ru-doped LiFeAs exhibit no imaginary frequencies across the Brillouin zone, confirming their dynamic stability and the absence of structural instabilities. A detailed analysis of the phonon dispersion and DOS in LiFeAs and LiFe$\phantom{0}_{0.5}$Ru$\phantom{0}_{0.5}$As is essential for understanding the underlying mechanisms of superconductivity in these systems. Such investigations not only shed light on the role of lattice dynamics but also support the design and optimization of materials with enhanced electronic and superconducting properties, as supported by previous studies^65^.

**Fig. 10:**
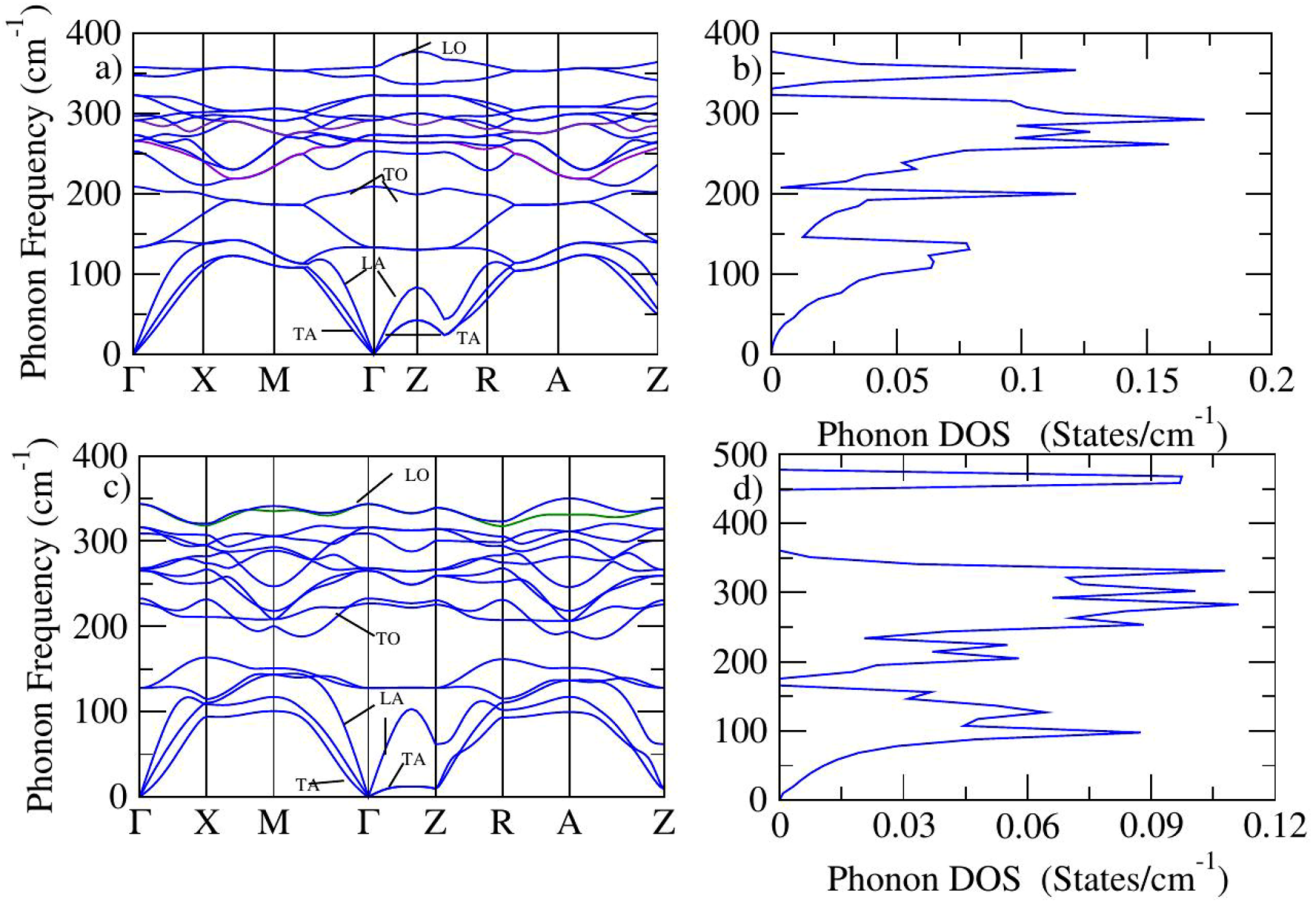
Phonon frequency of LiFeAs (**a**), phonon DOS of LiFeAs (**b**)^66^, Phonon frequency of 50% (**c**), phonon DOS of 50% (**d**) Ru doped LiFeAs.

In the phonon dispersion of LiFeAs, Fig. 10a, three acoustic branches are visible emerging from the $\Gamma$-point. These include two transverse acoustic (TA) modes and one longitudinal acoustic (LA) mode. The TA modes represent shear vibrations and are located at lower frequencies, while the LA mode involves compressional vibrations and appears at a slightly higher frequency. Near the $\Gamma$-point, these acoustic modes show a linear dispersion, indicating a stiff lattice with high phonon group velocities. The lighter atoms, such as Li and As, predominantly contribute to the higher frequency optical modes, whereas Fe atoms are involved in the lower frequency optical vibrations. The dispersion reveals a moderate separation between acoustic and optical branches, suggesting relatively weak phonon scattering and moderate electron-phonon interaction. The phonon density of states Fig. 10b, the acoustic region is dominated by contributions from Fe atoms due to their significant mass and bonding role in the lattice. In the mid-frequency both Fe and As atoms contribute, indicating mixed vibrational modes. At higher frequencies, the vibrations are mainly due to Li and As atoms, particularly in optical phonon branches. The smooth and well-distributed nature of the DOS confirms the dynamic stability of LiFeAs, as no imaginary frequencies are present.

In contrast, the phonon dispersion for 50% Ru-doped LiFeAs (Fig. 10c) shows noticeable modifications. The acoustic branches exhibit slightly lower slopes, indicating a reduction in sound velocity due to the introduction of heavier Ru atoms, which soften the lattice. The optical branches in the doped compound are more compressed and slightly shifted to lower frequencies compared to the pristine. This downward shift can be attributed to the heavier mass of Ru atoms, which replace Fe and thus lower the natural vibrational frequencies of the lattice. The phonon DOS of the Ru-doped system (Fig. 10d) reflects low-frequency acoustic region becomes more pronounced, with sharper peaks indicating an increased number of low-energy vibrational states. The introduction of Ru atoms primarily affects the low to mid-frequency regions, leading to a redistribution of vibrational states and potentially altering the thermal and electronic transport behavior.

### Fermi surface of LiFeAs and LiFe$\phantom{0}_{0.5}$Ru$\phantom{0}_{0.5}$As

The Fermi surface of LiFeAs via DFT methods confirms the presence of multiple Fermi pockets and provides insights into the role of electronic correlations. These features are consistent with the theoretical results reported^67,68^, suggesting moderate electronic correlations. Analyzing the Fermi surface and charge density of pristine and Ru-doped LiFeAs provides valuable insights into their electronic and superconducting properties.

**Fig. 11:**
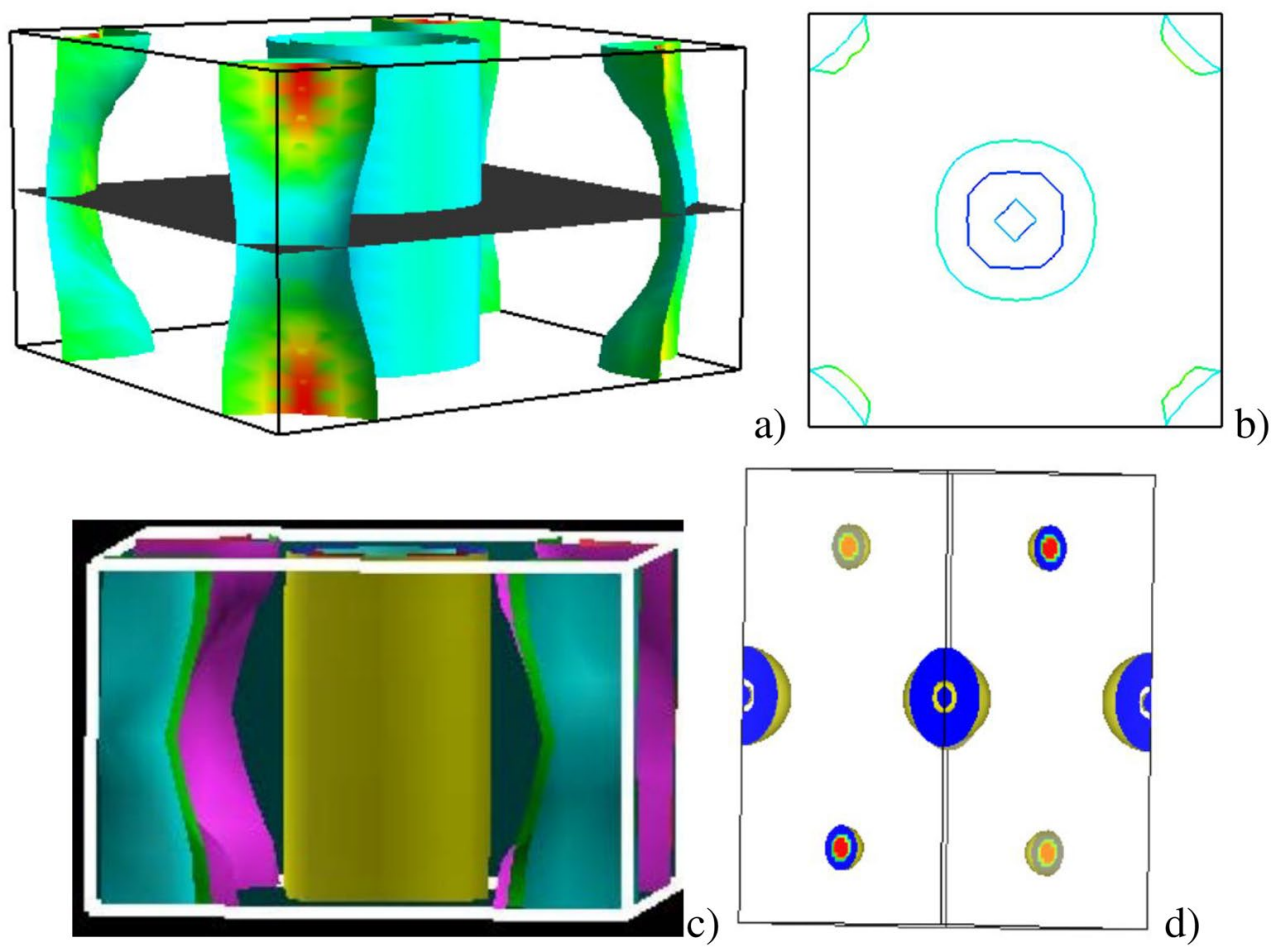
The Fermi surface of pristine LiFeAs (**a**), charge density of pristine LiFeAs (**b**), Fermi surface of 50% Ru-doped LiFeAs (**c**), and charge density of 50% Ru-doped LiFeAs (**d**). All plots were generated using FermiSurfer, version 2.4 https://fermisurfer.osdn.jp/.

Figure 11 shows the Fermi surface and charge density distributions of pristine and 50% Ru-doped LiFeAs. In pristine (Fig. 11a), the Fermi surface (FS) displays multiple hole pockets centered at the $\Gamma$ point and electron pockets near the M-point, consistent with the well-established multiband nature of Fe-based superconductors. This quasi-two-dimensional FS topology is favorable for nesting interactions, which are believed to enhance superconducting pairing via spin fluctuations^69^. The corresponding charge density map in Fig. 11b reveals strong electron localization around Fe and As atoms, indicative of significant covalent bonding within the Fe–As layers. Such bonding plays a critical role in stabilizing the lattice and facilitating efficient carrier mobility, which underpins the metallic and superconducting character of LiFeAs. Upon 50% Ru substitution, Fig. 11c, the FS undergoes substantial topological changes. The previously well-defined hole pockets at the $\Gamma$ point become less distinct, and the electron pockets appear more three-dimensional and diffuse. This suggests a partial suppression of FS nesting, likely diminishing the strength of interband scattering channels essential for unconventional superconductivity. Additionally, the FS becomes more isotropic, signaling a shift from the quasi-2D electronic character toward a more 3D-like electronic structure. The charge density distribution in Fig. 11d supports this evolution, showing a more delocalized electronic cloud around the Ru sites. This redistribution arises from the weaker electronegativity and more extended 4*d* orbitals of Ru compared to Fe, which disrupts the localized Fe–As covalent bonding network. As a result, hybridization between Fe/Ru and As states is weakened, leading to broader energy bands and reduced electronic correlations. These changes correlate with a reduction in the DOS at the Fermi level, observed in our separate DOS analysis, and may indicate a weakening of nesting-driven electronic instabilities.

## Conclusion

A first-principles investigation of Ru-doped LiFeAs was carried out using DFT and DFT+*U* methods to examine its electronic structure and magnetic properties. The optimized lattice parameter of 3.76 Å shows excellent agreement with the experimental value of 3.77 Å, validating the computational approach. The DFT+*U* method more effectively captures the localized nature of Fe-3*d* electrons, particularly at low Ru concentrations, whereas standard DFT tends to overdelocalize these states and underestimate magnetic interactions. With increasing Ru substitution, the system undergoes a clear transition from localized Fe-3d dominated states to more itinerant Ru-4d dominated states, most evident at 50% and 100% doping. Theoretical estimates of the Néel transition temperatures further emphasize the role of electron correlation: for 50% Ru-doped LiFeAs, DFT predicts $T^{(MFA)}$ = 80.15 K and $T^{(corr)}$ = 53.43 K, while DFT+*U* yields substantially higher values, $T^{(MFA)}$ = 325.3 K and $T^{(corr)}$ = 195.2 K, reflecting the stronger magnetic interactions captured by the Hubbard correction. In addition, Ru doping shifts the Fermi level upward and increases the binding energy per atom, indicating enhanced thermodynamic stability. The gradual crossover from correlated, localized Fe-3*d* states to delocalized Ru-4*d* states highlights the tunability of this system and reinforces its potential as a platform for advancing the understanding of superconductivity and correlated electron phenomena.

For future work, employing advanced many body techniques and hybrid approaches such as DFT+B, EPW-based electron–phonon coupling analysis, and beyond DFT methods would provide deeper insights into correlation effects and superconductivity in transition-metal–doped LiFeAs, especially with the computational power of CHPC resources.

## Data Availability

The data will be available from the corresponding author upon request.
